# DUBs, New Members in the Hypoxia Signaling clUb

**DOI:** 10.3389/fonc.2016.00053

**Published:** 2016-03-09

**Authors:** Amelie S. Schober, Edurne Berra

**Affiliations:** ^1^Centro de Investigación Cooperativa en Biociencias, CIC bioGUNE, Derio, Spain; ^2^Faculty of Health and Life Sciences, Center for Cell Imaging, Institute of Integrative Biology, University of Liverpool, Liverpool, UK

**Keywords:** DUBs, ubiquitination, HIF, protein homeostasis, cancer

## Abstract

Cellular protein homeostasis is tightly regulated by ubiquitination. Responsible for target protein ubiquitination is a class of enzymes, the so-called ubiquitin E3 ligases. They are opposed to a second class of enzymes, called deubiquitinating enzymes (DUBs), which can remove polyubiquitin chains from their specific target proteins. The coaction of the two sets of enzymes allows the cell to adapt its overall protein content and the abundance of particular proteins to a variety of cellular and environmental stresses, including hypoxia. In recent years, DUBs have been highlighted to play major roles in many diseases, including cancer, both as tumor suppressors and oncogenes. Therefore, DUBs are emerging as promising targets for cancer-cell specific treatment. Here, we will review the current understanding of DUBs implicated in the control of hypoxia-inducible factor, the regulation of DUBs by hypoxia, and the use of DUB-specific drugs to target tumor hypoxia-signaling.

## Introduction

Like most other posttranslational modifications (PTMs), ubiquitin (Ub) conjugation is a reversible modification (1). Ub E3 ligases covalently attach monomers of Ub to lysine (and also cysteine) residues of their target proteins. Furthermore, ligases also convert monoubiquitination into polyubiquitin chains by attaching one by one further Ub monomers to one of the seven internal lysine residues (K6/K11/K27/K29/K33/K48/K63) of the preceding Ub molecule. In contrast, the family of DeUBiquitinating enzymes (DUBs) breaks down those mono- and polyubiquitin chains from the target protein. Besides counteracting the action of the Ub E3 ligases, DUBs are proteases that process Ub precursors.

Of the nearly 100 DUBs encoded by the human genome, 79 are predicted to be active and mostly cleave particular types of Ub chain linkages from their respective target proteins. DUBs can be grouped into six families based on sequence and structure similarity: ubiquitin-specific proteases (USPs) that comprise the largest and most diverse subfamily, ubiquitin carboxyl-terminal hydrolases (UCHs), ovarian tumor proteases (OTUs), Josephins, JAB1/MPN/MOV34 (JAMMs), and the more recently discovered monocyte chemotactic protein-induced proteins (MCPIP). With the exception of JAMMs, which belong to the Zn^2+^-dependent metalloproteases, all the rest use the classical cysteine protease triad in the catalytical side (2).

Classically, the reversal of the polyubiquitination protects the target protein from being degraded by the proteasome, but ubiquitination has also been shown to have a broad range of non-catabolic functions (3). Thus, it is not surprising that DUB activity or inappropriate expression impacts on the regulation of multiple biological processes and several signaling pathways that are frequently altered in many disorders from cancer over neurodegenerative pathologies to inflammatory diseases [for more details, please refer to Ref. (4)]. Because of their direct or indirect implications in those diseases and because of their potential druggability, DUBs have become of increasing interest in recent years.

Hypoxia is a feature of most human cancers (5). The cancer cells and their environment adapt to and survive under low oxygen availability. The activation of the hypoxia-inducible factor (HIF) that orchestrates the hypoxia-signaling pathway is instrumental to this adaptation. HIF is a heterodimeric transcription factor that consists of a constitutively expressed β-subunit (HIF-β) and HIF-α, whose expression is tightly regulated through the ubiquitin-proteasome system (UPS) (6–8). HIF triggers the expression of hundreds of direct target genes, indirect transcription factors, and non-coding RNAs that enable cancer cell survival and tumor progression by promoting, among others, angiogenesis, metabolic rewiring, genomic instability, drug resistance, and the self-renewal capacity of cancer stem cells. HIFs are also activated by genetic alterations in human cancers, such as the von Hippel–Lindau protein (pVHL) loss of function in clear-cell renal carcinoma (9). Accordingly, sustained expression of HIF-α in tumors has been associated with higher aggressiveness, migratory, and metastasis-initiating potential and therefore worse prognosis (10, 11).

In this review, we will summarize the current knowledge about the action of DUBs on HIF-α and the regulation of those enzymes by hypoxia. We will also discuss the potential of exploiting DUBs to target tumor hypoxia signaling.

## The Canonical HIF Signaling Pathway

The adaptive cellular program in response to low oxygen availability is mainly triggered by two HIF-α subunits (HIF-1α and HIF-2α), which share several common targets but also exhibit non-redundant functions (12). Anyhow, the levels of both HIF-α subunits result from the dynamic interplay between their ubiquitination and deubiquitination. In well-oxygenated cells, HIF-α is very unstable, as it is degraded by the proteasome within approximately <5 min after translation, whereas HIF-α’s half-life is greatly increased in hypoxia (7, 8, 13). Proteasomal degradation is triggered by the continuous polyubiquitination of HIF-α by pVHL (9). pVHL is part of an E3 ligase complex and binds to HIF-α after the hydroxylation of two designated proline residues in HIF-α’s oxygen-dependent degradation domain (ODDD), the central regulatory domain that confers its oxygen sensibility (8). This binding can be stabilized by SSAT2, therefore enhancing HIF-α ubiquitination (14). The family of prolyl hydroxylase domain-containing proteins (PHDs), the oxygen sensors also referred to as EGLNs or HPHs, catalyze the hydroxylation of HIF-α (Pro^402^ and Pro^564^, in the case of HIF-1α) (15–19). HIF-α also harbors an N-terminal basic helix-loop-helix (bHLH) domain that mediates HIF-binding to the target DNA after heterodimerization with HIF-β/ARNT via the adjacent PAS domain. Of the two transactivation domains (TAD), the N-terminal TAD (N-TAD) lies within the ODDD, while the C-terminal TAD (C-TAD) is responsible for the recruitment of CBP/p300 needed to successfully induce the transcription of the HIF target genes that are characterized by having one or more HREs (hypoxia response elements) (20, 21). This C-TAD contains an asparagine residue (Asn^803^, in the case of HIF-1α) that upon oxygen-dependent hydroxylation by FIH (factor inhibiting HIF) hinders the successful interaction of HIF with CBP/p300 and therefore, HIF’s transactivation activity is reduced (22). Interestingly, HIF induces the expression of two of its negative regulators, PHD2 and PHD3, in order to ensure its own rapid degradation upon reoxygenation (19, 23). However, in conditions of chronic hypoxia, once the transcriptional adaptive program has been triggered, HIF-α levels drop again to avoid sustained HIF signaling and assure cell survival (24).

**Figure 1:**
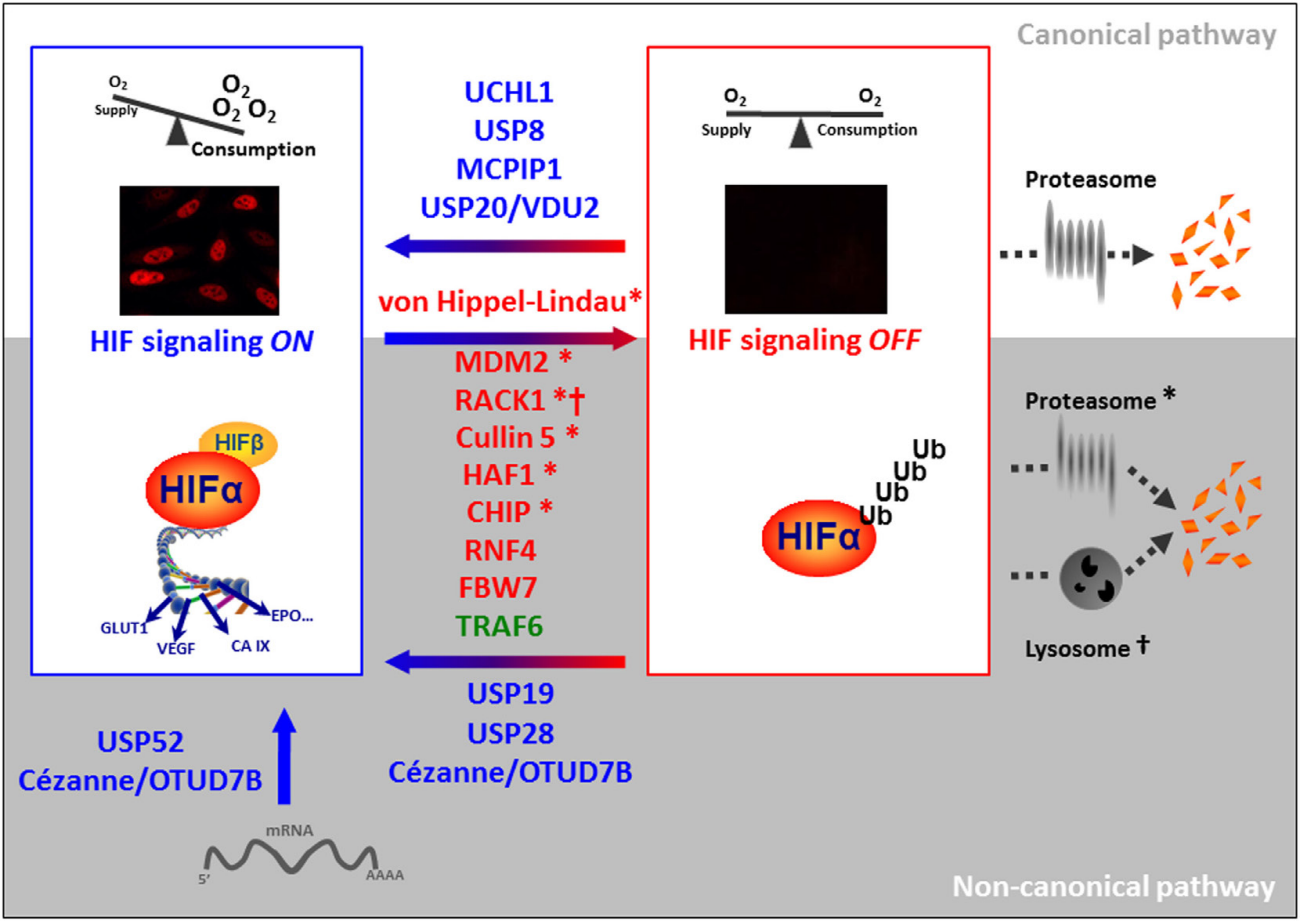
**Involvement of ubiquitinating and deubiquitinating enzymes in the regulation of HIF-α**. Destabilizing and stabilizing E3 ligases targeting HIF-α are pictured in red and green, respectively and DUBs are depicted in blue. E3 ligases that have been described to target HIF-α for proteasomal degradation are marked with a *; ^†^ refers to lysosomal HIF-α degradation.

In the context of the canonical HIF signaling pathway, so far there are relatively few DUBs reported in the literature, and reports are mostly focused on the impact on HIF-1α (Figure 1 upper part). *USP20* (also called pVHL interacting DUB2, VDU2) was the first DUB to be described to reverse pVHL-mediated HIF-1α ubiquitination (25). In turn, USP20 is a pVHL target (26). *MCPIP1* also deubiquitinates HIF-1α to promote angiogenesis (27). In the context of ciliogenesis, *USP8* has been found to bind to HIF-1α’s PAS domain and to partially protect HIF-1α from degradation (28). More recently, *UCHL1* has been shown to be a positive regulator of HIF-1α protein stability acting on HIF-1α’s ODDD (29).

## The Non-Canonical HIF Signaling

Not surprisingly because of HIF’s crucial role in cell fate, many more proteins have been described to be involved in the control of its stability (Figure 1 lower part). The heat-shock protein 90 (HSP90) that interacts with the PAS domain of HIF-α regulates its degradation in an O_2_/PHD/pVHL-independent manner (30). HSP90 competes with RACK1 for binding to HIF-α and prevents the recruitment of the elongin C/B Ub E3 ligase complex (31). A similar mechanism has been proposed for HIF-α activation by ErbB4 (32). As for other HSP90 client proteins, Cullin5 also regulates HIF-α degradation independently of elongin C/B function (33). The tumor suppressors p53, TAp73, and pTEN promote the Ub-mediated degradation of HIF-1α via recruitment of the Ub E3 ligase Mdm2 (34–36). Furthermore, Fbw7 ubiquitinates and induces HIF-1α degradation following phosphorylation by GSK3β (37, 38). Interestingly, this degradation can be antagonized by the Ub-specific protease (*USP28*) (38). Until now, this is the only non-canonical Ub E3 ligase–DUB pair identified for proteasomal degradation of HIF-α. HAF, the hypoxia-associated factor, seems to play a dual role in the control of HIF-α stability and/or activity. While HAF acts as an Ub E3 ligase targeting HIF-1α for degradation independently of oxygen availability, hypoxia-induced SUMOylated HAF promotes HIF-2α transactivation without affecting its stability (39, 40). Furthermore, RNF4 controls the levels of SUMOylated HIF-2α (41). *USP19* seems to be required for the hypoxic accumulation of HIF-1α, though the effect is not dependent on its deubiquitinase activity (42). USP19 is further substrate of Siah-1 and Siah-2 Ub E3 ligases, which also control the stability of PHD1, PHD3, and FIH (43–45). Thus, further studies are necessary to clarify the direct impact of USP19 in HIF-1α ubiquitination.

The chaperone-dependent Ub ligase CHIP targets HIF-1α but not HIF-2α for degradation either by the proteasome or by the autophagic machinery, the second big protein degradation and recycling pathway that has been implicated in the elimination of ubiquitinated HIF-α (46–49). In this regard, *Cezanne* (OTUD7B), a deubiquitinase targeting K11 Ub chains (50), has been reported to protect HIF-1α from lysosomal degradation. While this process is independent of HIF-1α prolyl hydroxylation, it depends on the presence of pVHL (51).

Calpain and the activation of the forkhead transcription factor FOXO4 destabilize HIF-α, although the underlying molecular mechanisms are unknown (52, 53). Further studies are also needed to characterize the role of Parkin in the regulation of HIF-α, based on its identification within the Parkin-dependent ubiquitinome by a proteomic approach (54). In contrast with all the previous reports, it is worth mentioning the role played by the Ub E3 ligase TRAF6. TRAF6 increases HIF-1α but not HIF-2α polyK-63 ubiquitination and protects the protein from proteasomal degradation (55).

In addition to HIF-α stability, mRNA expression and activity of the transcriptional complex fine-tune HIF regulation. In this regard, *USP52* is required for the protection of HIF-1α (but not HIF-2α) mRNA from premature degradation and therefore, allows the normal hypoxic induction of HIF-1α (56). The case of USP52 is somewhat special as this protein, although structurally related to the family of USPs, lacks the catalytic cysteine (57). Besides protecting HIF-1α protein from its degradation, *Cezanne*’s catalytical activity is also required for maintaining basal levels of the E2F1 transcription factor. Moniz et al. demonstrated that E2F1 controls the expression of HIF-2α mRNA and therefore, established an indirect role of the DUB Cezanne in HIF-2α expression (58).

Finally, a number of DUBs have been shown to regulate transcription factors and signaling pathways that cross talk with HIFs, likely contributing to the complexity and specificity of the cellular hypoxic response, even though they go beyond the scope of this review (59–61).

## Regulation of DUBs by Hypoxia

As for other enzymes, there are several possible layers of regulation of DUB activity. Next to the transcriptional regulation, the stability and translation of the mRNA can be regulated by mRNA-processing enzymes. The turnover and therefore, the availability of the mature protein can be set by a variety of PTMs. PTMs can also interfere with the binding of the DUBs to their target proteins or other interactors, as well as modulate reversibly and irreversibly the (auto) catalytical activity of the DUB. Hypoxia, being an extreme cellular stress condition, should be able to regulate deubiquitinating activity on all the possible different layers in order to adapt DUB functions to the cell’s needs. However, the literature about the regulation of specific DUBs by hypoxia (1% O_2_, if not specified differently) is still scarce and almost exclusively restricted to transcriptional regulation. For instance, the expression of *USP13* is reduced upon treatment with as little as 6 h of 2% O_2_ in melanoma cell lines (59). The reduction of the mRNA also translates to the protein level and causes the loss of Siah-2 stabilization. Similarly, in colon cancer cells hypoxia reduces *USP46* mRNA and protein levels and, therefore, diminishes USP46’s stabilizing effect on the tumor suppressors PHLPP1 and PHLPP2, conferring to the colon cancer cells an increased paclitaxel resistance (62, 63). Guo et al. provide more detailed information about the hypoxia-mediated transcriptional regulation of the UCH *CYLD*. They suggest that the decrease of CYLD mRNA and protein seen in glioblastoma cells is due to the hypoxia-induced increase of the transcriptional repressors Snail and Hes1 (64). In contrast, hypoxia has been shown to increase *Cezanne* via p38 MAPK (65).

An et al. claimed that CYLD is targeted for proteasomal degradation after interaction with the HPV E6 protein in hypoxia (66). This is to date the only report of a posttranslational regulation of DUB activity by hypoxia. However, Lee et al. present evidence that the activity of many, if not most, DUBs depends on the redox state of the cell. They show that the catalytically active cysteine residue can be oxidized, for instance, by intracellular hydrogen peroxide, leading to the abolishment of the deubiquitinating activity. The inactivating oxidation can be reversed in the presence of reducing agents, such as DTT, or prevented by antioxidants (67). As hypoxia and mitochondrial ROS production are intrinsically linked it might not be too far-fetched to propose that hypoxia directly modulates DUB activity via ROS.

## Dysregulation of Hypoxia-Related DUBs in Cancer

Given the importance of Ub-mediated changes in protein function and homeostasis, it is not by chance that the entire process is highly regulated. Disruption of the ubiquitination cycle by mutations or altered expression of specific components within the cascade has been associated with several disorders. In particular, more than 30 DUBs have been associated with cancer directly or indirectly. Both, the loss of a specific DUB activity or its hyperactivity are non-desired events if the targets are tumor suppressors or oncogenes, respectively. Recurrent mutations of DUBs are rare in cancer with only few exceptions. Gene fusions with RUNX are reported for USP42 and USP16 in hematologic diseases, such as chronic myelomonocytic leukemia and acute myeloid leukemia. However, dysregulated mRNA levels of DUBs are implicated in many malignancies. Here, we will focus only on a few examples of hypoxia-related DUBs, for a more extensive overview please refer to the very comprehensive review by D’Arcy et al. (68).

Germline mutations of the tumor-suppressor gene *CYLD* are prevalent in familial cylindromatosis, a genetic condition that leads to predisposition for developing multiple skin tumors (69, 70). In addition, CYLD deubiquitinating activity has been seen to be abolished in different cancers on the protein level by inactivating phosphorylations or destabilizing polyubiquitination (71). More recently, it has been reported that *USP8* is frequently mutated in adenomas causing Cushing’s disease (72).

*USP28* is a DUB whose overexpression has been reported in breast and colon cancer and glioblastoma (73, 74). A recent publication has proposed USP28 to be a potential predictive marker in bladder cancer, as they found correlation of USP28 with tumor histological grade, clinical stage, recurrence, and survival (75). Similar to USP28, *UCHL1* has also been proposed to be a useful biomarker, being overexpressed in gastric cancer (76) and in myeloma (77), and epigenetically down-regulated in colorectal cancer (78). As mentioned above, downregulation of *USP46* may serve as a biomarker of resistance to chemotherapy in colon cancer (63). Finally, despite being inconsistent to its role in the regulation of HIF-1α and HIF-2α, decreased *Cezanne* expression is associated with the progression and poor prognosis in hepatocellular carcinoma (79).

## DUBs as Druggable Targets for Therapy

Modulators of individual UPS components are emerging as a novel class of anticancer drugs. The initial research focus had been directed toward targeting the proteasome, with activity described for many compounds with proteasome inhibitory activity, including bortezomib. Because Ub E3 ligases provide substrate specificity, their direct targeting may avoid the deleterious side effects associated with the global inhibition of the proteasome, making them interesting candidates as drug targets. Nutlin-3 and JNJ-26854165 are classic examples directed against the Ub E3 ligase MDM2 and are currently undergoing clinical evaluation as anticancer therapy.

Newly arising, DUBs may serve as equally or more useful targets. Indeed, DUBs are highly specialized and evolutionary linked to proteases, a typified pharmaceutical target class for drug discovery, thanks to their well-characterized catalytical domain. Several partial and specific inhibitors against a small number of DUBs have been developed, have proved active in preclinical studies as reviewed recently by D’Arcy and Linder (80), and have provided feasibility for targeting these enzymes for anticancer purposes. Among them, HBX 41,108 is a partially selective USP inhibitor because it inhibits USP5, USP7, USP8, and UCHL3 in addition to caspase 3 (81). This is to our knowledge the only DUB inhibitor so far described as targeting one of the DUBs linked to the HIF signaling pathway. Interestingly, the inhibition of USP8 suppresses growth of gefitinib-resistant non-small cell lung cancer cells, though no link to the potential impact on HIF-1α is reported (82). It is tempting to speculate about new drugs directed against hypoxia-related DUBs that succeed to fight intratumoral hypoxia-signaling in the coming years.

## Conclusion

HIF-α protein homeostasis is tightly controlled in healthy cells in order to avoid inappropriate activation of HIF signaling. A variety of E3 ligases and DUBs are involved in this task by triggering and protecting HIF-α from its degradation, respectively. Permanent activation of the HIF signaling pathway has been found in many tumors and seems to be beneficial for tumor growth and cancer progression. In most cases, the reason for sustained HIF-α protein levels in the tumor cells are still not revealed, but a possible mechanism is the pathological increase of HIF-α specific DUB activity. In recent years, the dysregulation of deubiquinating enzymes in cancer (and other diseases) has become of increasing interest, and alterations of their expression and activities have been shown to have diagnostic value. Whether cancer-related events that lead to the upregulation of DUB activity are the primary cause of uncontrolled HIF signaling, or whether initial hypoxia upregulates DUB expression as a positive feed-back-loop is not determined. But in the light of DUBs being druggable enzymes, it is important to understand their implications in HIF and tumor hypoxia-signaling.

## Author Contributions

ASS and EB contributed with review writing, editing, and final approval of the manuscript.

## Conflict of Interest Statement

The authors declare that the research was conducted in the absence of any commercial or financial relationships that could be construed as a potential conflict of interest.
